# Fasting increases the phosphorylation of AMPK and expression of sirtuin1 in muscle of adult male northern elephant seals (*Mirounga angustirostris*)

**DOI:** 10.14814/phy2.13114

**Published:** 2017-02-27

**Authors:** Debby Lee, Bridget Martinez, Daniel E. Crocker, Rudy M. Ortiz

**Affiliations:** ^1^ Department of Cellular and Molecular BiologyUniversity of CaliforniaMercedCalifornia; ^2^ Department of BiologySonoma State UniversityRohnert ParkCalifornia

**Keywords:** Deiodinase, insulin, lipid metabolism, PGC1*α*, thyroid hormone

## Abstract

Fasting typically suppresses thyroid hormone (TH)‐mediated cellular events and increases sirtuin 1 (SIRT1) activity. THs may regulate metabolism through nongenomic pathways and directly through activation of adenosine monophosphate‐activated protein kinase (AMPK). Adult male elephant seals (*Mirounga angustirostris*) are active, hypermetabolic, and normothermic during their annual breeding fast, which is characterized by stable TH levels. However, the contribution of TH to maintenance of their fasting metabolism is unknown. To investigate the fasting effects on cellular TH‐mediated events and its potential association with SIRT1 and AMPK, we quantified plasma TH levels, mRNA expressions of muscle SIRT1 and TH‐associated genes as well as the phosphorylation of AMPK in adult, male northern elephant seals (*n* = 10/fasting period) over 8 weeks of fasting (early vs. late). Deiodinase type I (DI1) expression increased twofold with fasting duration suggesting that the potential for TH‐mediated cellular signaling is increased. AMPK phosphorylation increased 61 ± 21% with fasting suggesting that cellular metabolism is increased. The mRNA expression of the TH transporter, monocarboxylate transporter 10 (MCT10), increased 2.4‐fold and the TH receptor (THr*β*‐1) decreased 30‐fold suggesting that cellular uptake of T_4_ is increased, but its subsequent cellular effects such as activation of AMPK are likely nongenomic. The up‐regulation of SIRT1 mRNA expression (2.6‐fold) likely contributes to the nongenomic activation of AMPK by TH, which may be necessary to maintain the expression of PGC‐1*α*. These coordinated changes likely contribute to the up‐regulation of mitochondrial metabolism to support the energetic demands associated with prolonged fasting in adult seals.

## Introduction

Adult male northern elephant seals (NES) (*M  angustirostris*) experience a 2–3 month period of prolonged food deprivation as part of their life history and have the highest sustained rates of fasting energy expenditure compared to other male pinnipeds (Crocker et al. 2012b). During this fasting period, adult male NES are simultaneously competing for territory to establish access to estrus females and defending harems (Haley et al. 1994; le Boeuf 1974), losing about 36% of their arrival body mass (Crocker et al. 2012b; le Boeuf and Laws 1994). Although such prolonged periods with absolutely no food or water consumption could have detrimental effects on energy balance in most mammals, adult NES have evolved robust physiological mechanisms to cope with these conditions. However, the factors contributing to the potential regulation of cellular metabolism during this energy‐demanding fast remains largely underexplored.

For several terrestrial species, prolonged fasting is characterized by down‐regulation of metabolic rate via suppression of cellular thyroid hormone (TH)‐mediated events and TH levels to conserve energy (Araujo et al. 2009; Azizi 1978; Azizi et al. 1979; Diano et al. 1998; Kohrle 2000; McMillin et al. 1980; Oppenheimer et al. 1987; Vella et al. 2011). THs are key regulators of metabolism and are known to be correlated with body weight and energy expenditure (Fox et al. 2008; Iwen et al. 2013; Knudsen et al. 2005). The traditional mechanism for TH action involves the outer ring deiodination of thyroxine (T_4_) to the more cellularly active, 3,5,3′‐triiodothyronine (T_3_) (Mullur et al. 2014; van Heyningen and Glaysher 2012), through deiodinase type I (DI1) or type II (DI2). Deiodination of T_4_ by type III (DI3) (and to a much lesser extent, DI1) produces reverse T_3_ (rT_3_), which suppresses metabolism and protects the organism from energetic burden during reduced energy intake (Diano et al. 1998; Lopresti et al. 1991; St. Germain 1994). When T_4_ is mono‐deiodinated, the resulting T_3_ can bind to its tissue‐specific nuclear receptor such as TH receptor beta‐1 (THr*β*‐1) to initiate the transcription of several genes, which occurs over hours to days (Yen 2001; Zhang and Lazar 2000).

THs have also been shown to regulate metabolism through nongenomic effects, which are not dependent on nuclear receptor‐mediated T_3_ actions (Cheng et al. 2010). Exogenously infused T_3_ and T_4_ can act through extranuclear plasma membrane receptors or transporters on a timescale of minutes (Davis et al. 2008), providing a nongenomic mechanism for TH signaling independent of traditional nuclear receptor‐mediated signaling (Yonkers and Ribera 2009). Moreover in nongenomic signaling, T_4_ has a higher binding affinity than T_3_ and may directly interact with integrin cell membrane receptors (Yonkers and Ribera 2009). Monocarboxylate transporter 10 (MCT10) is a specific thyroid hormone transporter that effectively facilitates transmembrane transport of both free T_4_ and T_3_ (Friesema et al. 2003), and is highly expressed in skeletal muscle (van der Deure et al. 2010). In transfected cells that express DI1, DI2, and DI3, MCT10 effectively facilitated both the cellular uptake and efflux of TH resulting in an increase in intracellular metabolism (Friesema et al. 2008, 2006).

Similar to THs, sirtuin 1 (SIRT1) is also a major regulator of energy metabolism (Cohen et al. 2004; Kanfi et al. 2008). SIRT1 is a NAD+‐dependent deacetylase that is triggered by adenosine monophosphate‐activated protein kinase (AMPK) during nutrient or energy deprivation (Canto et al. 2010; Fulco et al. 2008). The nuclear protein, peroxisome proliferator‐activated receptor coactivator‐1‐alpha (PGC‐1*α*), facilitates the AMPK‐mediated activation of SIRT1, and serves as a cofactor involved in both TH and SIRT1 downstream signaling (Attia et al. 2010; Rodgers and Puigserver 2007). For example, during short‐term exercise and endurance training, muscle PGC‐1*α* is readily inducible (Baar et al. 2002; Goto et al. 2000; Norrbom et al. 2004; Russell et al. 2003) and is essential to increasing mitochondrial oxidative metabolism (Czubryt et al. 2003; Lin et al. 2005). Activation of SIRT1 stimulates mitochondrial fatty acid oxidation genes to promote insulin sensitization (Gerhart‐Hines et al. 2007) and gluconeogenesis (Rodgers and Puigserver 2007). Peroxisome proliferator‐activated receptor gamma (PPARγ) also contributes to cellular metabolism by regulating whole‐body glucose homeostasis and insulin sensitivity (Amin et al. 2010; Lee et al. 2006; Rieusset et al. 1999), and is regulated by SIRT1 (Pardo and Boriek 2011; Picard et al. 2004).

The physiological adaptation to prolonged fasting is commonly associated with the suppression of TH production (Boelen et al. 2008) and higher activity in SIRT1 (Kanfi et al. 2008; Rodgers et al. 2004). While TH and SIRT1 share common target genes especially in lipid and fasting metabolism (Hashimoto et al. 2001; Li et al. 2007; Ness et al. 1990; Rodgers et al. 2004), the association between TH and SIRT1 during prolonged food deprivation remains poorly defined. In fasted mice, it has been suggested that an increase in the SIRT1 protein requires the fasting‐associated suppression of TH action including the inactivation of the THr*β*‐1 (Cordeiro et al. 2013). Although this highlights the potential link between TH and SIRT1, the mechanism behind this signaling pathway is poorly understood. TH function has important implications in northern elephant seal health and disease (Yochem et al. 2008). In fasting adult northern elephant seals, THs levels are unchanged (Crocker et al. 2012b); however, studies examining SIRT1 activity in mammals that undergo a natural and prolonged fasting period are lacking. Therefore, to elucidate the mechanisms regulating the cellular function of TH and its potential association with SIRT1, we quantified the mRNA expression of TH‐associated genes, SIRT1, PPARγ, MCT10, and the TH/SIRT1 target gene, PGC‐1*α*, and the phosphorylation of AMPK. We hypothesized that prolonged fasting decreased TH‐associated genes and increased SIRT1 in adult male NES.

## Methods

All procedures were reviewed and approved by the Institutional Animal Care and Use Committees of both the University of California, Merced and Sonoma State University. All work was conducted under the National Marine Fisheries Service marine mammal permit #87‐1743.

### Animals

Ten individual adult male northern elephant seals (*M. angustirostris*) were studied at Año Nuevo State Reserve, CA during their natural breeding fast. Each male was sampled at two periods, which we refer to as “early” fasting (initial measurement) and “late” fasting (52 ± 4 days after initial measurement) for the purpose of this paper. The early fasting values served as the control or the point of reference from which the fasting‐associated changes were assessed.

### Sample collection and preparation

Males were chemically immobilized by intramuscular injection of ~0.3 mg kg^−1^ teletamine HCl/Zolazepam HCl (Telazol, Fort Dodge Animal Health, Fort Dodge, IA). Blood samples were obtained through an 18 g spinal needle placed in the extradural vein and placed on ice until transport to the laboratory. Blood samples were centrifuged at 4°C and stored at ‐80°C until analysis. Muscle biopsies were obtained from the *latissimus dorsi* as described previously (Araujo et al. 2008; Martinez et al. 2013; Vazquez‐Medina et al. 2010). Muscle biopsies were rinsed with cold, sterile saline, placed in cryogenic vials, immediately frozen in liquid nitrogen, and stored at −80°C until analysis.

### Body mass and daily energy expenditure

Body mass and composition were estimated and daily energy expenditure (DEE) was calculated as described previously (Crocker et al. 2001, 2012a,b). Briefly, a comprehensive set of morphometric measurements was combined with 16 blubber ultrasound measurements and the truncated cone method was used to calculate loss of adipose and lean tissue between measurements. These tissue losses were converted to metabolizable energy to estimate DEE over the measurement period.

### Quantification of mRNA expressions

Total RNA was isolated from muscle samples using TRIzol reagent (Invitrogen, Carlsbad, CA) following the manufacturer's instructions. The RNA integrity was confirmed by measuring the absorbance at 260 nm/280 nm and by running bands on a 1% agarose gel electrophoresis (Sambrook and Russell 2006). Contamination of genomic DNA in total RNA was eliminated by digestion with DNase I (Roche, Indianapolis, IN) as specified by the manufacturer. Isolated cDNAs from muscle were synthesized from total DNA‐free RNA (1 *μ*g) using oligo‐dT and the QuantiTect Reverse Transcription kit (Qiagen, Valencia, CA).

Specific primers for DI1, DI2, DI3, THr*β*‐1, PGC‐1*α*, and glyceraldehyde 3‐phosphate dehydrogenase (GAPDH) were previously designed for elephant seals (Martinez et al. 2013, 2016). Primers for SIRT1, PPARγ, and MCT10 were designed based on homologous mammalian nucleotide sequences (Table 1). To confirm primer amplification, and verify results of PCR reactions, products were run through an electrophoresis gel and visualized under UV light. Gene expression was measured by real‐time quantitative PCR (RT‐qPCR) using DI1Fw + DI1Rv, DI2Fw + DI2Rv, DI3Fw + DI3Rv, THr*β*‐1Fw + THr*β*‐1Rv, PGC‐1*α* Fw + PGC‐1*α*Rv, PPARγ Fw + PPARγ Rv, MCT10Fw + MCT10Rv and GAPDHFw + GAPDHRv primers, respectively. The RT‐qPCR reactions and sample analyses were performed as previously described in our hands (Martinez et al. 2013, 2016). Positive and negative controls were included for each reaction where negative controls were reactions without cDNA and positive controls were reactions using the RT product of each specific gene. Each gene was amplified, run on a gel, and the estimated molecular weights of the products were used to confirm the utility of the primers. The expression of GAPDH was used as an internal standard to normalize the expression of each target gene. Previous studies from our laboratory have confirmed that GAPDH expression is an appropriate and utile gene for normalizing other genes because its expression does not change with fasting duration (Martinez et al. 2013, 2016), which was also confirmed in this study.

**Table 1:** Primers designed to obtain the cDNA sequences of elephant seal sirtuin 1 (SIRT1), peroxisome proliferator‐activated receptor gamma (PPARγ), and monocarboxylate transporter 10 (MCT10)

Primer name	Nucleotide sequence (5–3)
SIRT1Fw	CCCAGCTGAACCACTTGC
SIRT1Rv	GAGGCACTTCATGGGGTATGG
PPARGFw	CAGAAGTGCCTTGCT
PPARGRv	GGTCAGCGGGAAGGA
MCT10Fw	GCTTTACTTACCGACCTCTTG
MCT10Rv	CCAGGCACATAATCTGCAATCC

### Quantification of protein expression by western blotting

Protein expression was semi‐quantified by standard western blot as previously described (Vazquez‐Medina et al. 2010, 2011, 2012; Viscarra et al. 2011a,b, 2012). The primary antibodies for p‐AMPK (Thr‐172; sc‐33524) and AMPK (sc‐74461) (Santa Cruz Biotechnology, Santa Cruz, CA) were diluted 1:250. Secondary antibodies (C40721‐02 [p‐AMPK] and C40910‐04 [AMPK]; Li‐Cor Biosciences, Lincoln, NE) were diluted 1:20,000. Membranes were blocked in Li‐Cor blocking buffer for 1 h with gentle shaking at room temperature, and then were incubated overnight with the primary antibody in Li‐Cor blocking buffer and 0.2% Tween‐20. After incubation, membranes were washed and incubated with the secondary antibody containing Tris‐buffered saline containing 0.2% Tween‐20 and 5% fat‐free milk, and rewashed. Blots were visualized and analyzed using Odyssey Clx, Li‐Cor Imager (Li‐Cor Biosceinces, Lincoln, NE). For each sample, the same amount of total protein (50 *μ*g) was loaded per well to help normalize the quantification. The ratio of p‐AMPK to AMPK was used to assess degree of phosphorylation, and thus, activation.

### Plasma analyses

The plasma concentrations of thyroid stimulating hormone (TSH) and the THs, free T_4_ (fT_4_), total T_4_ (tT_4_), free T_3_ (fT_3_), total T_3_ (tT_3_), and reverse T_3_ (rT_3_), were measured by radioimmunoassay (Siemens, Washington, DC; Alpco, Salem, NH), and previously validated for elephant seals (Ensminger et al. 2014; Ortiz et al. 2003, 2001). All samples were analyzed in duplicate and run in a single assay with intra‐assay percent coefficients of variability of <5% for all assays. Plasma glucose was measured in duplicate using an auto‐analyzer (YSI 2300, Yellow Springs, OH).

### Statistics

Means (± S.E.M.) for early fasting were compared to late fasting by repeated measures analysis of variance and were considered significantly different at *P *< 0.05 to assess the fasting‐associated changes. Paired t‐test were performed for all plasma analyses. Becauase the muscle biopsy samples were limiting and not always paired (early and late samples), full complementary analyses were not possible, and were therefore analyzed by unpaired t‐test. The relationships between DEE and hormones were compared using linear regression analysis and were considered significantly different at *P *< 0.05. Because tT_4_ and rT_3_ did not change significantly between the early and late fasting periods, the two values for each animal were averaged, and this averaged hormone value was used in the regression analyses with DEE. Because fT_3_ changed significantly between the two sampling periods, the change in value (Δ fT_3_) was used in the regression analysis with DEE. Statistical analyses were performed using R 2016: A Language and Environment for Statistical Computing (Vienna, Austria).

## Results

### Fasting effects on body mass and energy expenditure

Body mass and daily energy expenditure (DEE) were measured to provide a functional context for comparisons to changes in hormones and cellular events. Mean body mass decreased 29% (*P *< 0.01) between early (1559 ± 87 kg) and late (1103 ± 58 kg) fasting. Mean DEE was 183 ± 50 MJ day^−1^ between the two sampling periods.

### Prolonged fasting increased free T_4_ and T_3_, and decreased total T_3_

**Figure 1:**
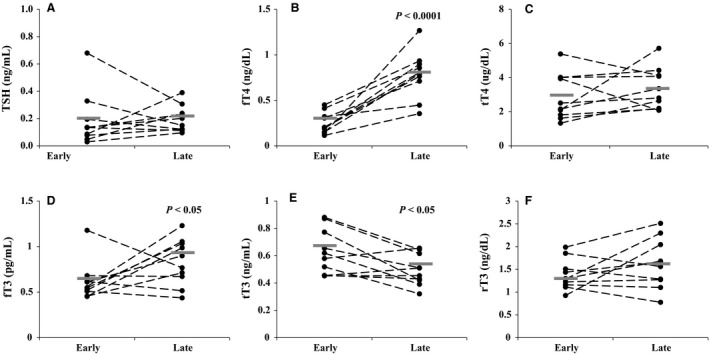
Paired plasma concentrations of (A) TSH, (B) free thyroxine (fT_4_), (C) total T_4_ (tT_4_), (D) free tri‐iodothyronine (fT_3_), (E) total T_3_ (tT_3_), and (F) reverse T_3_ (rT_3_) for each animal between early and late fasting. Mean values for early and late samples are denoted by a gray bar. The degree of significance is denoted by the *P*‐value directly on the figure. TSH, thyroid stimulating hormone.

To assess the circulating levels of TH between early and late fasting in adult NES, free and total TH levels as well as rT_3_ were measured. Fasting increased mean fT_4_ (*P *< 0.0001) and mean fT_3_ (*P *< 0.05), and decreased (*P *< 0.05) mean tT_3_ (Fig. 1). However, mean TSH, rT_3_, and tT_4_ did not change (Fig. 1).

### Fasting increased the potential for TH‐associated nongenomic effects

**Figure 2:**
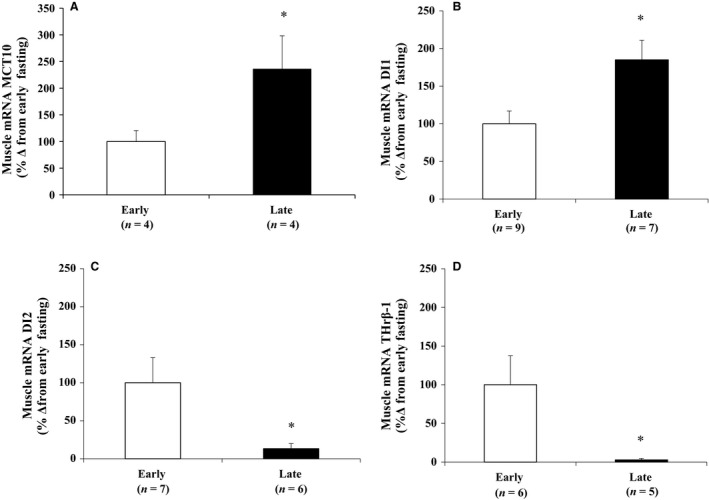
Mean (± S.E.M.) mRNA expressions of (A) monocarboxylate transporter 10 (MCT10), (B) deiodinase type I (DI1), (C) type II (DI2), and (D) thyroid hormone receptor beta‐1 (THr*β*‐1) as percent change from early. * denotes significantly different (*P* < 0.05) from early

The mRNA expression of MCT10 was measured to evaluate the potential for cellular uptake of T_4_ and T_3_. Mean muscle MCT10 increased (*P *< 0.05) 2.4‐fold with fasting (Fig. 2A).

The mRNA expression of DI1, 2, and 3 were measured to evaluate the potential for converting cellular THs between early and late fasting. Fasting increased the muscle mRNA expression of DI1 (*P *< 0.05) nearly twofold (Fig. 2B), but decreased the expression of DI2 (*P *< 0.05) approximately fourfold (Fig. 2C). Muscle mRNA expression of DI3 was undetectable suggesting that rT_3_ levels generated locally were likely the result of DI1. To assess the potential for inducing changes in gene expression, the mRNA expression of THr*β*‐1 was measured. Muscle mRNA expression of THr*β*‐1 decreased (*P *< 0.05) approximately 30‐fold between early and late fasting (Fig. 2D).

### Prolonged fasting increased the potential for elevated cellular metabolism

**Figure 3:**
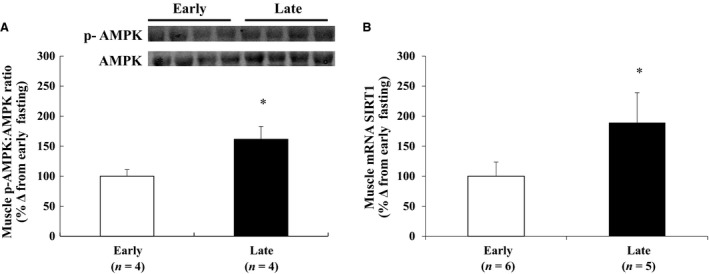
Mean (± S.E.M.) (A) ratio of the protein expression of p‐AMPK to AMPK, *inset* is representative western blot, and (B) mRNA expression of sirtuin 1 (SIRT1) as percent change from early. *denotes significantly different (*P* < 0.05) from early

To assess the effect of fasting on the energy state of muscle in adult male northern elephant seals, the phosphorylation of AMPK and mRNA expression of SIRT1 were measured. The phosphorylation of AMPK (p‐AMPK to AMPK) increased (*P *< 0.05) 61 ± 21% (Fig. 3A), and the expression of SIRT1 mRNA increased (*P *< 0.05) 2.6‐fold between early and late fasting (Fig. 3B).

### Prolonged fasting decreased mRNA expression of PPARγ, but not PGC‐1*α*

**Figure 4:**
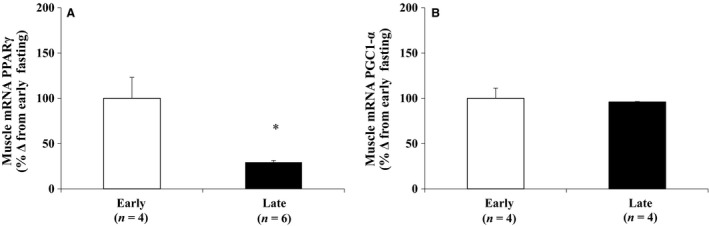
Mean (± S.E.M.) mRNA expressions of (A) peroxisome proliferator‐activated receptor gamma (PPARγ), and (B) peroxisome proliferator‐activated receptor coactivator 1‐alpha (PGC‐1*α*). * denotes significantly different (*P* < 0.05) from early

To assess the potential functionality of the decrease in THr*β*‐1 and increase in AMPK activity with fasting duration, the expressions of muscle PPARγ and PGC‐1*α* were measured, respectively. Mean muscle PPARγ expression decreased (*P *< 0.05) 3.3‐fold with fasting (Fig. 4A), but the expression of PGC‐1*α* was maintained between early and late fasting (Fig. 4B).

### Prolonged fasting does **not** appear to induce an insulin‐resistant phenotype

To better assess the relationship between alterations in TH‐mediated signaling and glucoregulatory metrics, plasma glucose, insulin, and adiponectin were measured. Mean plasma glucose (10.5%) and insulin (13%) decreased (*P *< 0.05) between early and late fasting, which is not indicative of an insulin‐resistant phenotype (Table 2). This is further supported by the lack of a change in plasma adiponectin (Table 2).

**Table 2:** Mean ± (S.E.M.) plasma adiponectin, insulin, and glucose from adult male northern elephant seals (*n* = 10) during early and late fasting duration

	Early	Late
Adiponectin (ng/mL)	234 ± 8	248 ± 13
Insulin (*μ*U/mL)	2.01 ± 0.06	1.76 ± 0.09^a^
Glucose (mmol/L)	7.49 ± 0.26	6.70 ± 0.24^a^

a Denotes significant difference (*P *< 0.05) from early.

### DEE changes accordingly with tT_4_, fT_3_, and rT_3_

**Figure 5:**
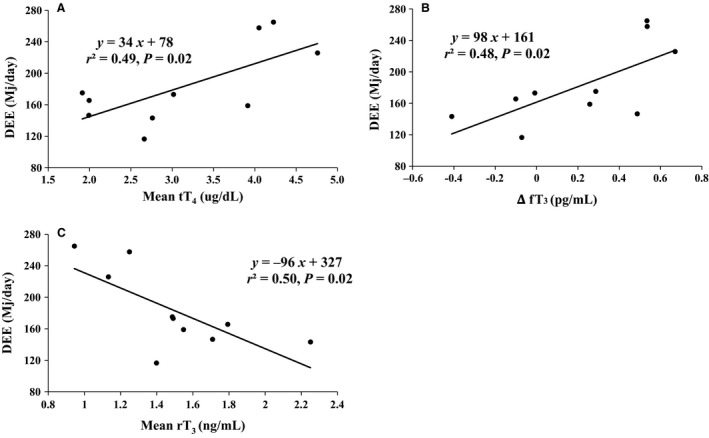
Relationships between daily energy expenditure (DEE) and (A) mean total thyroxine (tT_4_), (B) change in free tri‐iodothyroxine (ΔfT_3_), and (C) mean reverse T_3_ (rT_3_). Correlations were considered significant at *P* < 0.05.

To assess the relationships between DEE and THs, we performed linear regressions between the two variables. DEE was positively related to mean tT_4_ (y = 34 x + 78; *r* ^*2* ^= 0.49; *P* = 0.02) (Fig. 5A) and change in fT_3_ (y = 98 x + 161; *r* ^*2* ^= 0.48; *P* = 0.02) (Fig. 5B). Conversely, DEE was inversely related to mean rT_3_ (y = −96 x + 327; *r* ^*2* ^= 0.50; *P* = 0.02) (Fig. 5C).

## Discussion

Suppression of TH levels, DI1 and DI2 activity, TH receptor, and reciprocal increases in DI3 and rT_3_ are typical responses to prolonged food deprivation in most mammals to conserve energy (Araujo et al. 2008; Azizi 1978; Diano et al. 1998; Kohrle 2000; Oppenheimer et al. 1987; Vella et al. 2011). However, adult male NES exhibit relatively high metabolic rates despite their fasting state (Crocker et al. 2012b). This would appear to be paradoxical, given that we have previously demonstrated that total T_4_ and T_3_ levels were unchanged with fasting duration in adult male NES (Crocker et al. 2012b). Interestingly, the relative difference in TH levels between fasting pups and adult males suggest that a higher rate of T_4_ deiodination is needed to support the increase in energy expenditure in adult males (Crocker et al. 2012a; Martinez et al. 2013, 2016). Thus, this apparent paradox would suggest that circulating plasma levels are insufficient to properly access the cellular activity and metabolism that would be expected to support the relatively high metabolic rates of adult NES. Therefore, this study was performed to more robustly assess the potential contribution of TH‐mediated signaling on mechanisms of cellular metabolism during prolonged fasting in adult male seals.

Initially, we examined the potential for TH‐mediated effects to alter cellular metabolism through changes in the expression of related genes. Among the most intriguing data revealed with respect to the TH‐mediated events are: (1) the robust suppressions of DI2 and THr*β*‐1, and (2) the near doubling of MCT10. The decreases in DI2 and THr*β*‐1would suggest that the potential of TH‐mediated changes in gene expression is greatly reduced, and thus, argue for more nongenomic effects to support cellular metabolism. The increase in MCT10 would support this contention as the rapid influx of primarily T_3_ could initiate effects on the order of minutes as opposed to the much slower genetic effects mediated by THr*β*‐1 (Davis et al. 2008; Sinha and Yen 2000). The increases in plasma fT_4_ may be the consequence of decreased DI2 as the conversion of T_4_ to T_3_ would be reduced. Additionally, the reduction in THr*β*‐1 could contribute to an increase in fT_3_, which is the intracellularly available form of the hormone to bind the receptor. Furthermore, the increase in muscle DI1 expression is likely a compensatory change to the decrease in DI2 to allow for, or at least the maintenance of, T_4_ deiodination locally. The strong, positive correlations between average tT_4_ and the change in fT_3_ with DEE suggest that the dynamic changes in T_4_ and T_3_ pools may contribute to energy expenditure in fasting adult seals. In further support of this contention is the lack of an increase in rT_3_ and the undetectable levels of muscle DI3 expression. These observations in plasma rT_3_ and DI3 suggest that the need to suppress cellular metabolism via rT3 effects is abrogated. The strong, inverse relationship between average rT_3_ and DEE provides corroborating support for the suggestion that the decrease in the rT_3_‐mediated suppression of cellular metabolism may facilitate energy expenditure during fasting. While it is well recognized that plasma rT_3_ levels are primarily the result of hepatic secretion, the undetectable levels of muscle DI3 suggests that, at least locally, the generation of local rT_3_ is unnecessary. If muscle is under similar thyroidal regulation as the liver, the unchanging concentrations of rT_3_ suggests that DI1 is preferentially deiodinating T_4_ to T_3_, and not to rT_3_. Collectively, these synchronized changes work in concert to contribute to cellular metabolism, which translates to changes in daily energy expenditure in fasted, adult seals.

The regulation of cellular metabolism by T_3_ can also be achieved by its contribution to the activation of AMPK via the transcriptional increase in THr*β*‐1 expression (Wang et al. 2014). However, we demonstrated that in prolong‐fasted, adult seals, THr*β*‐1 is almost completely down‐regulated despite increased DI1 levels (and presumably the increased deiodination of T_4_) suggesting that the potential regulation of AMPK by thyroid hormones in adult male NES is not likely through classical mechanisms that alter gene expression. This is supported by studies demonstrating that T_3_ may nongenomically regulate metabolism via phosphorylation and activation of kinase pathways and proteins including muscle AMPK during fasting (Branvold et al. 2008; Irrcher et al. 2008). Given that these adult male NES are hypermetabolic (Crocker et al. 2012b), all‐the‐while actively mating and fighting while simultaneously fasting, we suggest that the increase in fT_3_ coupled with increased DI1 and MCT10 are necessary to support the nongenomic activation of AMPK. The advantage of such a system for energetically burdened mammals is that it would provide a quicker and more efficient stimulation of TH‐AMPK cellular signaling and minimizing the lengthy, genetic transcription pathways that are more metabolically costly (Sinha and Yen 2000).

The nongenomic actions of TH begin at the plasma membrane and require a receptor or transporter to enter the cell (Nishimura and Naito 2008; visser 2000). MCT10, which is able to transport both T_4_ and T_3_, is widely expressed in many tissues, with particularly high expression in skeletal muscle (Nishimura and Naito 2008), and appears to transport T_3_ better than T_4_ (visser 2000). The increased mRNA expression of MCT10 suggests that the rapid, nongenomic effects could be facilitated by the influx of THs into the cell, and may contribute to the activation of AMPK to support the cellular metabolism of male seals throughout their fast.

In fasted mice, increased SIRT1 expression depended on TH suppression and involved the inactivation of THr*β*‐1 (Cordeiro et al. 2013). Replacement of T_4_ attenuated the incremental increase of SIRT1 suggesting that this inverse relationship is an integrated metabolic response to fasting, as the increase in SIRT1 requires the fasting‐associated suppression of TH secretion (Cordeiro et al. 2013). While that may be the case here as well, we alternatively suggest that the increase in SIRT1 was also orchestrating the suppression of THr*β*‐1, which would enable the quicker, nongenomic, TH‐mediated activation of AMPK. This scenario is corroborated by the fact that SIRT1 may bind directly to DNA‐bound transcription factors including nuclear receptors to influence transcriptional activity independent of THr*β*‐1during fasting conditions (Kemper et al. 2009; Li et al. 2007; Suh et al. 2013; Wilson et al. 2010).

Both AMPK and TH may regulate the expression of PGC‐1*α*, which is typically increased by fasting (Liang and Ward 2006). The activation of AMPK increases PGC‐1*α* expression (Kanoh et al. 2001; Yonkers and Ribera 2009), and AMPK requires PGC‐1*α* activity to modulate the expression of several key contributing factors in mitochondrial and glucose metabolism (Li 2013). Furthermore, SIRT1 also regulates metabolic processes by deacetylating important transcriptional regulators including PGC‐1*α*, and also has the capacity to impair PPARγ (Canto and Auwerx 2009; Dominy et al. 2010; Pardo and Boriek 2011). The data suggest that the increase in AMPK phosphorylation may be necessary to just maintain the expression of PGC‐1*α* (and likely PGC‐1*α* activity). The maintenance of PGC‐1*α* expression is likely independent of a TH‐mediated process here given that THr*β*‐1 was dramatically reduced.

Fasting or low caloric diet may improve insulin sensitivity (Kanoh et al. 2001), and is associated with a decrease in the expression of PPARγ in adipose of obese humans (Vidal‐Puig et al. 1997). However, the effects of prolonged fasting on the expression of PPARγ in skeletal muscle is not well defined. In this study, the decrease in the mRNA expression of PPARγ suggests that the sensitivity of muscle to insulin is reduced, which is consistent with the decrease in plasma insulin and lack of change in plasma adiponectin. While collectively this suite of changes is not indicative of fasting‐induced insulin resistance, it does suggest that insulin‐dependent glucose utilization is suppressed to help ameliorate fasting‐induced hypoglycemia. Furthermore, the decrease in plasma glucose and insulin are consistent with the increase in AMPK phosphorylation (Canto and Auwerx 2009; Canto et al. 2010; Hardie et al. 2012; Ruderman et al. 2013).

**Figure 6:**
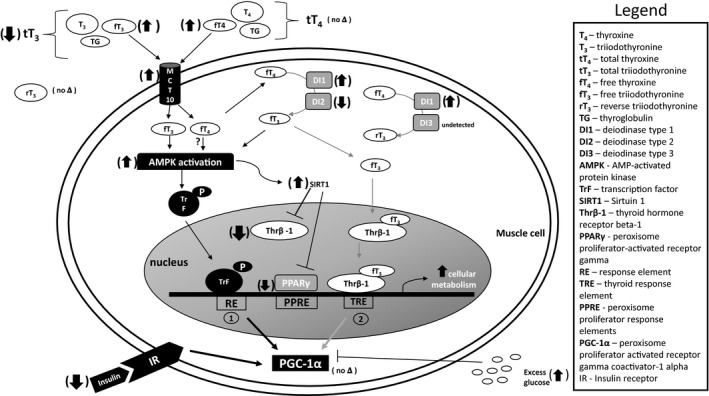
Suggested mechanism of triiodothyronine (T_3_)‐induced gene expression in muscle of prolong‐fasted adult male northern elephant seals. (1) The more rapid thyroid hormone (TH)‐mediated, nongenomic pathway includes the activation of kinases such as AMPK by T_3_ and the suppression of TH receptor beta‐1 (THr*β*‐1) by sirtuin 1 (SIRT1). AMPK phosphorylates and activates transcription factors (TrF), which maintain peroxisome proliferator‐activated receptor coactivator 1‐alpha (PGC‐1*α*) in the presence of reduced insulin and glucose. (2) The slower, classical genomic mechanism of T_3_‐induced gene expression involves the binding of T_3_ to TH receptors such as THr*β*‐1. This mechanism causes an exchange of cofactor proteins and increases TH receptor‐mediated transcription to increase cellular metabolism. Thick dark arrows show the changes found in adult male northern elephant seals during fasting duration. Thin black arrows suggest nongenomic mechanisms while thin gray arrows suggest genomic mechanisms of TH during prolonged fasting.

Despite the correlative and descriptive nature of the results, which limit their interpretative value, this study reveals some novel insight to the potential regulation of TH‐associated cellular metabolism in an adult mammal naturally adapted to prolonged fasting while maintaining relatively high levels of energy expenditure. The increases in the free fractions of TH, the expressions of DI1 and MCT10, and the activation of AMPK in the presence of near complete down‐regulation of THr*β*‐1 are suggestive of nongenomic mechanisms of TH‐associated changes in cellular metabolism (Fig. 6). We propose that such a mechanism in seals may provide a more efficient process to activate AMPK and SIRT1 to support the seal's relatively high metabolism. While previous studies demonstrated that an increase in SIRT1 with fasting requires the suppression of TH secretion, muscle SIRT1 expression in this study increased with fasting despite increased circulating levels of free T_4_ and T_3_, and the lack of suppressed total levels. We propose that regardless of the levels of circulating thyroid hormones, it is the availability of the TH receptor that needs to be suppressed to permit the up‐regulation of SIRT1 to punctually allow THs to nongenomically activate AMPK in a mammal that is naturally adapted to tolerate excessive energetic demands. By allowing both T_4_ and T_3_ into the cell, MCT10 may play a role in the increase of muscle AMPK activation. Furthermore, the increase in AMPK phosphorylation may be necessary to maintain the expression of PGC‐1*α*, which further contributes to the up‐regulation of mitochondrial metabolism to support the energetic demands associated with prolonged fasting in adult seals. The decrease in the mRNA expression of PPARγ suggests that the sensitivity of muscle to insulin is reduced, which is consistent with the decrease in plasma insulin. Overall, these results may provide a more profound understanding of the regulation of cellular metabolism that highlights the intricate interactions among AMPK, SIRT1, and the changes in the expression of genes that regulate metabolism in mammals that have physiologically evolved to tolerate periods of absolute deprivation of food and water all‐the‐while maintaining a hypermetabolic state.

## Author Contributions

D.L., B.M., D.E.C., and R.M.O. participated in the conception and design of research; D.L. and D.E.C. performed experiments; D.L., B.M., D.E.C., and R.M.O. interpreted results of experiment; D.L. analyzed data, prepared figures, and drafted manuscript; D.L., B.M., D.E.C, and R.M.O. edited and revised manuscript; D.L., B.M., D.E.C, and R.M.O approved final version of manuscript.

## Conflict of Interest

No competing interests, financial or otherwise, are declared by the authors.
